# Sonic Hedgehog Induces Mesenchymal Stromal Cell Senescence-Associated Secretory Phenotype and Chondrocyte Apoptosis in Human Osteoarthritic Cartilage

**DOI:** 10.3389/fcell.2021.716610

**Published:** 2021-09-27

**Authors:** Meng Feng, Wenguang Liu, Jing Ding, Yusheng Qiu, Qian Chen

**Affiliations:** Department of Orthopedics, Rhode Island Hospital, Warren Alpert Medical School of Brown University, Providence, RI, United States

**Keywords:** osteoarthritis, sonic hedgehog (SHH), mesenchymal stromal (stem) cell (MSC), chondrocyte, senescence associated secretory phenotype (SASP), cartilage

## Abstract

Hedgehog (HH) signaling plays a critical role in osteoarthritis (OA) pathogenesis, but the molecular mechanism remains to be elucidated. We show here that Sonic Hedgehog (*SHH*) gene expression is initiated in human normal cartilage stromal cells (NCSC) and increased in OA cartilage mesenchymal stromal cells (OA-MSCs) during aging. Manifesting a reciprocal cellular distribution pattern, the SHH receptors *PTCH1* and *SMO* and transcription factors *GLI2* and *GLI3* are expressed by chondrocytes (OAC) in OA cartilage. SHH autocrine treatment of osteoarthritis MSC stimulates proliferation, chondrogenesis, hypertrophy, and replicative senescence with elevated SASP gene expression including *IL1B*, *IL6*, *CXCL1*, and *CXCL8*. SHH paracrine treatment of OAC suppresses *COL2A1*, stimulates *MMP13*, and induces chondrocyte apoptosis. The OA-MSC conditioned medium recapitulates the stimulatory effects of SHH on OAC catabolism and apoptosis. *SHH* knock-down in OA-MSC not only inhibits catabolic and senescence marker expression in OA-MSC, but also abolishes the effect of the OA-MSC conditioned medium on OAC catabolism and apoptosis. We propose that SHH is a key mediator between OA-MSC and OA chondrocytes interaction in human OA cartilage *via* two mechanisms: (1) SHH mediates MSC growth and aging by activating not only its proliferation and chondrogenesis, but also low-grade inflammation and replicative senescence, and (2) SHH mediates OA-MSC-induced OAC catabolism and apoptosis by creating a pro-inflammatory microenvironment favoring tissue degeneration during OA pathogenesis.

## Introduction

Osteoarthritis (OA) is an aging-related disease of the entire joint, including cartilage matrix breakdown, synovial hyperplasia, subchondral bone sclerosis, and remodeling (Koelling et al., 2009; Lepage et al., 2019). It causes joint pain, muscle atrophy, and functional disability (Glyn-Jones et al., 2015). As the prevalence of OA increases with age, joint cartilage aging has an alarming impact on the life quality of aging populations, who bear an enormous cost and societal burden for OA treatment (Jafarzadeh and Felson, 2018; Liu et al., 2018). However, developing therapeutics to halt aging-related OA, containing healthcare costs, and improving geriatric life quality are challenging because of a lack of understanding of the patho-physiological mechanisms of OA (Vincent, 2020). The cellular and molecular basis of cartilage aging is an area of active investigation. The signal transduction pathways leading to uncontrolled activation of the sterile inflammation in articular cartilage during aging are yet to be understood.

An increasing number of senescent cells accumulate within aged cartilage, undermining cartilage integrity and increasing the risk of OA pathogenesis (Jeon et al., 2017). Telomere shortening occurs in chondrocytes during aging and results in cellular senescence, a permanent form of growth arrest with increased expression of the senescent markers *p21* and *p16^ INK4a^* (Fellows et al., 2017; Diekman et al., 2018; Faust et al., 2020). While the density of chondrocytes in articular cartilage reduces during aging, the number of cartilage-resident mesenchymal stromal cells increases during the development of OA (Fellows et al., 2017; Jayasuriya et al., 2018). However, specific deletion of *p16^*INK*4a^*-positive chondrocytes did not inhibit the increased production of senescence-associated secretory phenotype (SASP) factors during aging (Diekman et al., 2018). Therefore, identification of specific senescent cells in joint cartilage and understanding of the mechanism by which they reach senescence during aging are essential for the development of OA therapy.

In addition to chondrocytes, cartilage mesenchymal stromal cells (MSCs, chondroprogenitor cells, *aka.* CPCs) exist in articular cartilage (McGonagle et al., 2017; Jayasuriya et al., 2019; Grandi et al., 2020). CD166-positive MSCs can be isolated from normal young adult cartilage (normal cartilage stromal cells, *aka* NCSC) (Jayasuriya et al., 2019) and aged OA cartilage (OA mesenchymal stromal cells, OA-MSC) (Jayasuriya et al., 2018). While NCSCs are capable of differentiating into chondrocytes in response to cartilage damage *in vivo* (Koelling et al., 2009; Seol et al., 2012), OA-MSCs play a vital role in regulating cartilage tissue regeneration and degeneration during OA (Nelson et al., 2014; Jayasuriya et al., 2018). Although MSCs repopulated damaged cartilage after mechanical injury induction, they exhibited relatively high levels of chemokines (CXCL8 and CXCL12) and cytokine expression (interlukin-6, IL-6), and excessive metalloproteinase production (MMP1, MMP13, ADAMTS4) relative to chondrocytes (Seol et al., 2012; Zhou C. et al., 2014; Ji et al., 2019; Grandi et al., 2020). Since these molecules belong to the SASP family, we focused on the mechanisms of the induction of SASP gene expression in OA-MSC in this study.

The hedgehog protein family, including Sonic Hedgehog (SHH) and Indian Hedgehog (IHH), have been shown to play an important role during skeletal development and homeostasis (Alman, 2015; Zhu et al., 2020). While SHH is expressed by mesenchymal progenitor cells during limb bud patterning (Alman, 2015; Tan et al., 2018), IHH is expressed by pre-hypertrophic chondrocytes within the growth plate (Zhou J. et al., 2014). HH is a mitogen and morphogen, which not only induces mesenchymal progenitor cell proliferation, chondrogenesis, and bone formation during development but also promotes tumorigenesis as an oncogene (Dormoy et al., 2009; Bishop et al., 2010; Alman, 2015). Blocking hedgehog (HH) signaling attenuates cartilage degeneration and OA pathogenesis (Lin et al., 2009), although the precise molecular mechanism remains unclear. Canonical signaling of HH is mediated by a multi-component receptor complex that includes Patched homolog 1 (PTCH1), Smoothened (SMO), and the Gli family of transcription factors (Alman, 2015; Fang et al., 2020). While PTCH1 inhibits SMO and downstream transcription factors, the binding of HH to PTCH1 receptor releases the basal inhibition of SMO by PTCH1, thereby activating recipient cell activity (Woods et al., 2019). Since IHH is synthesized by pre-hypertrophic chondrocytes in developing cartilage, it is thought that IHH contributed to OA pathogenesis (Lin et al., 2009; Zhou J. et al., 2014; Woods et al., 2019). However, recombinant IHH does not cause extracellular matrix degradation during its incubation with healthy cartilage *in vitro* (Thompson et al., 2015), while other studies implicated SHH in OA pathogenesis (Lin et al., 2014; Akhtar et al., 2015).

In this study, we aimed to elucidate the signaling pathways of the interaction between chondrocytes (OACs) and OA-MSCs, two types of cells in human OA articular cartilage. We isolated primary OA-MSCs and OAC from human cartilage and utilized the newly generated OA-MSC line in our laboratory (Jayasuriya et al., 2018; Jayasuriya et al., 2019; Liu et al., 2020). Our results demonstrated that SHH rather than IHH drove human OA-MSC senescence and SASP induction, which resulted in OAC catabolism and apoptosis.

## Materials and Methods

### Ethics Statement

The study was approved by the Institutional Review Board of Rhode Island Hospital (approved project number: 002910-2013) in accordance with the declaration of Helsinki, and all patients were given their informed consent. Tibia plateau or femoral condyle cartilage samples were obtained from 18 patients with unicompartmental osteoarthritis (67.88 ± 1.623 years, 13 male and 5 female) post total knee arthroplasty at Brown Medical School affiliated hospitals.

### Cartilage Samples Collection: Inclusion and Exclusion Criteria

OA was diagnosed according to the American College of Rheumatology criteria, and rheumatoid arthritis was excluded (Altman et al., 1990). Clinical and radiological criteria were used for the classification of OA; patients with systemic inflammatory diseases such as rheumatoid arthritis were excluded. Cartilage tissue samples were kept at 4°C and rushed within 2 h to the laboratory for processing. Portions of cartilage with damaged articular surface (Outerbridge Grade 2–3) and smooth articular surface (Outerbridge Grade 0–1) were used for histological analysis (Williams et al., 2010; Liu et al., 2020).

### Propagation of Chondrocytes From Cartilage

Cartilage biopsies were taken from the unicompartmental osteoarthritic cartilage and scored for macroscopic disease severity using the Outerbridge classification (normal-looking cartilage, Grade 0; cartilage with softening and swelling, Grade 1; partial-thickness defect with superficial fissuring at the depth of 1–2 mm, Grade 2; deep fissuring, Grade 3; exposed subchondral bone, Grade 4). OA chondrocytes were isolated from the normal appearance area (Outerbridge Grade 0–1) and standardized in culture with similar OA scores (Jayasuriya et al., 2018). To standardize the procedure and to avoid contamination of the chondrocytes with bone marrow cells, the subchondral lamella was left intact. Cartilage slices were directly transferred into a dish containing HBSS, minced into fine fragments, and washed twice in PBS supplemented with antibiotics. The fragments were further digested in 10 ml of Type I A crude bacterial collagenase (Sigma-Aldrich, St. Louis, MO, United States) at a concentration of 1.0 mg/ml with serum-free high glucose DMEM (HyClone, Ottawa, ON, Canada) for 12 h in a 37°C shaking water bath. Cells were strained through a 100 μm nylon cell strainer (Thermo Fisher Scientific, Waltham, MA, United States) to remove clumps and washed three times with 5.0 ml of DMEM supplemented with 10% FBS. Cells were seeded in culture dish at an average density of 2 × 10^4^ cells/cm^2^, passaged once after 1 week at 90% confluence by 0.25% Trypsin EDTA treatment (Life Technologies, Logan, UT, United States) to eliminate matrix debris and cultured for maximally 1 more week in order to obtain sufficient number of cells. Media were changed three times a week. Those in the first through second passages were used for subsequent experiments.

### Cell Sorting, Enrichment, and Characterization of Cartilage-Derived Mesenchymal Stromal Cells

Briefly, cartilage-derived mesenchymal stromal cells were enriched using a previously described method using fibronectin (Jayasuriya et al., 2018; Liu et al., 2020); 6-cm dishes had been coated with 10 μg/ml of fibronectin at 4°C overnight prior to seeding 2,000 cells/ml at 37°C. After 20-min incubation, non-adherent cells were washed away. When single cells formed, individual colonies consisting of ≥32 cells were isolated and reseeded in individual wells of six-well cell culture plates. Establishment and characterization of OA-MSC cell lines and normal cartilage stromal cell (NCSC) lines were described previously (Jayasuriya et al., 2018, 2019). The OA-MSC and NCSC cell lines were found to retain the gene expression pattern and multi-lineage differentiation potentials of primary cell counterpart, respectively (Jayasuriya et al., 2018, 2019). The cartilage-derived OA-MSC and NCSC cell lines retain the MSC marker expression pattern and multi-lineage differentiation potentials similar to the primary MSC (Jayasuriya et al., 2018, 2019; Liu et al., 2020). OAMSCs were cultured in DMEM supplemented with 10% FBS, 1% Pen. Strep (Gibco, Life Technologies), 100 mM HEPES (Gibco), 2 mM *L*-glutamine (Gibco), 0.1 mM ascorbic acid (Thermo Fisher Scientific), 0.1 mM sodium pyruvate (Gibco), 0.5 mg/mL *L*-glucose (Gibco). Then primary OA-MSC as well as cell line at lower passages (from 2 to 7) was used for experiments.

### Sonic Hedgehog Stimulation

SHH N-Terminus with a 20 kDa molecular weight was purchased from R&D system (Cat#1845-SH-100; R&D Systems, Minneapolis, MN, United States); 0.1 × 10^6^ OA chondrocytes, primary OA-MSC, and OA-MSC line 5 were treated with recombinant human SHH in a 12-well plate for 24 h (5 μg/ml) (Akhtar et al., 2015).

### CCK-8 Proliferation Assay

One thousand OA-MSCs or chondrocyte suspensions (100 μl/well) were seeded in a 96-well plate and then incubated in the humidified incubator. A 10 μl cell counting kit-8 solution (Cat#96992, Sigma-Aldrich) were added into each well and the plate was incubated for 4 h at 37°C, 5% CO_2_ prior to detecting absorbance. Measurement of the absorbance at 450 nm was detected by a microplate reader (Spectramax M2 device; Molecular Devices, San Jose, CA, United States).

### Preparation of Osteoarthritis Mesenchymal Stromal Cell-Derived Conditioned Medium

Conditioned medium (CM) was collected from OA mesenchymal stem cells, then 0.1 × 10^6^ OA chondrocytes were cultured in a medium containing OA-MSC-conditioned medium at the ratio of 50:50 (volume/volume) in a 12-well plate for 24 h, as described elsewhere (Wang et al., 2017; Jayasuriya et al., 2019). Moreover, 0.1 × 10^6^ OA-MSCs were treated with target siRNA of SHH in a 12-well plate for 24 h then washed with HBSS three times, and normal growth medium was replaced for extra 24 h. siSHH-CM was collected from siRNA-treated OA-MSCs and added into OA chondrocytes. OA chondrocytes were incubated with conditioned medium at 37°C, allowing cells to uptake paracrine factors from OA-MSC before ultimate collection.

### RNA Purification, Sequencing, and Bioinformatics

For RNA-sequencing, total RNA of human OAC, NCSC, and OA-MSC was isolated and purified as described previously (Liu et al., 2020). Briefly, RNA sequencing was conducted by Genewiz, Inc. RNA-seq reads, analyses, and heatmap were generated as described previously (Liu et al., 2021).

### Quantitative Real-Time Reverse Transcriptase-Polymerase Chain Reaction

The total RNA was extracted using RNAiso plus (Takara, Shiga, Japan) according to the manufacturer’s instructions, and RNA concentration and purity were determined using a NanoDrop spectrophotometer; 500 μg RNA was used to synthesize complementary DNA (cDNA) using a reverse transcription reagents kit (Roche, Mannheim, Germany) based on the manual. The real-time quantitative PCR was carried out following the protocol and conducted with an Applied Life Technologies ProFlex PCR system. A reaction mixture containing the SYBR Green Master Mix (Qiagen, Hilden, Germany), and the appropriate primers were added to a 96-well plate, together with cDNA template, for a final reaction volume of 20 μl/well, and then following the temperature profile: run for an initial step at 95°C for 15 min, followed by 40 cycles of amplification at 94°C for 10 s, followed by 60°C for 30 s, and then 72°C for 15 s. Primers were used to evaluate Hedgehog families (*IHH*, *SHH*, and *DHH*), cell senescence markers (*p16^*INK*4a^/CDKN2a, p21*, *IL1B*, *IL6*, *CXCL1*, and *CXCL8*), chondrogenic markers (*SOX9* and *COL2A1*), hypertrophic chondrocyte marker (*COL10A1*), pro-inflammatory markers, matrix-degrading markers, and osteogenic marker (*RUNX2*) together with stable housekeeping gene 18S RNA. The gene expression was normalized to the housekeeping gene 18S RNA in the same sample. They were designed *via* Premier Version 5.0 (Premier Biosoft International, Palo Alto, CA, United States). The cycle threshold (*Ct*) values for 18S RNA and those of samples were measured and calculated by BioRad software. Fold expression was analyzed using the Ct method (relative transcript levels were calculated as *x* = 2^–Δ^ ^Δ^ *^*Ct*^*, where ΔΔ*C*_*t*_ = Δ*E* – Δ*C*, and Δ*E* = *C*_*t exp*_ – *C*_*t* 18 *s*_; Δ*C* = *C*_*t ctl*_ – *C*_*t* 18*s*_). Each sample was analyzed using two separate arrays, and values for replicate arrays were averaged.

### Western Blotting

OA-MSCs and OACs were collected and treated with 1 × radioimmuno-precipitation lysis buffer (Thermo Fisher Scientific) supplemented with Halt protease inhibitor (Cat#78430, Thermo Fisher Scientific). Cell lysis was then sonicated for 15 s, heated to 95°C for 5 min, and stored at −20°C until use for the sodium dodecyl sulfatepoly acrylamide gel electrophoresis; 20-μg protein samples were loaded and transferred to nitrocellulose membrane probed with anti-SHH antibody (Cat#2207, at 1:1,000 dilution, Cell Signaling Technology, Danvers, MA, United States), anti-SMO antibody (Cat#ab235183, at 1:500 dilution, Cambridge, MA, United States), anti-RUNX2 antibody (Cat# ab23981, at 1:5,000 dilution, Abcam), anti-SOX9 (Cat# ab185230, at 1:2,000 dilution, Abcam), and anti-MMP13 antibody (Cat#39012, at 1:3,000 dilution, Abcam) and normalized with β-Actin (Cat#ab108349, at 1:1,000 dilution, Abcam, United States). After the primary antibody incubation, the membranes were labeled with secondary anti-rabbit IgG antibody (at a dilution of 1:15,000, IRDye infrared dye, LI-COR Biosciences, Lincoln, NE, United States) and were then imaged using the Licor Odyssey CLx scanner (LI-COR Biosciences). Quantification of Western blot data by evaluating the gray values of protein expression level was performed using software in the Odyssey Infrared Imaging system.

### Small Interfering RNA

RNAi was performed in primary OA-MSCs and OA-MSC cell line. 0.1 × 10^6^ cells were seeded in a 12-well plate (Cat#150628, Thermo Fisher Scientific). Cells were transfected with 50 nM small interfering RNA (siRNA) targeted against SHH (ON-TARGETplus smart pool) or a non-targeting siRNA as the control (Dharmacon; Horizon, Lafayette, CO, United States). Gene expression was assessed by qPCR using cDNA synthesized from RNA extracted from each well, with five technical repeats per analyzed gene.

### Detection of Apoptosis

Primary chondrocytes were plated at the seeding density of 10^4^ cells per well and treated with SHH ligand or conditioned medium in a culture chamber with 4-well glass slide (Cat#354104, Corning Inc., Corning, NY, United States). We washed cells twice with 100 μl assay buffer by carefully pipetting up and down by using apoptosis detection kit (Cat#ab176749, Abcam), resuspended cells in 200 μl assay buffer, added 2 μl of apopxin green indicator (200×) to cells, and added 1 μl cytocalcein 450 (200×) stock solution to cells to detect live cells. We incubated cells at room temperature for 1 h, washed cells two times with 200 μl assay buffer, and replaced with clean 200 μl assay buffer. We analyzed cells under the fluorescence microscope using FITC channel for apoptosis indicator (Nikon digital imaging system, Tokyo, Japan).

### Histological Processing

Human OA cartilage samples were prepared and processed (4 mm in width, 10 mm in height, and 10 mm length) using a handsaw for histological analysis. Briefly, OA cartilage samples were divided at 1 cm × 0.4 cm × 1 cm, directly fixed in 4% paraformaldehyde in phosphate-buffered saline (PBS 1×, pH = 7.4) for 48 h, and then subjected to paraffin embedding. Sections were then de-paraffinized, re-hydrated in xylene and graded ethanol, and stored at 4°C.

### Immunofluorescence Staining

The cassettes with OA cartilage were sectioned at 6-μm thickness, mounted onto slides, heated at 58°C for 30 min, cleared with xylene twice, and rehydrated using sequential incubation in 100, 95, and 70% ethanol for 10 min, respectively. Antigen retrieval was performed by treating samples with HA and H_2_O_2_. After blocking with 10% goat serum, slides were incubated with the primary antibodies: anti-SHH (Cat#06-1106, 1:100, Millipore, Sigma), anti-SMO antibody (1:100, Abcam), and mouse anti-CD166 (Cat#MABN1785, 1:100, EMD Millipore) in antibody dilution buffer overnight at 4°C. Slides were then incubated with the secondary antibodies: goat anti-rabbit IgG Alexa Fluor 594 (Cat#ab150080, 1:200, Life Technologies) and donkey anti-mouse IgG H&L Alexa Fluor 488 (Cat#ab150105, 1:200, Abcam) in for 30 min. Finally, slides were mounted using anti-fade mounting medium with 4′-6-diamidino-2-phenylindole (DAPI). Negative control was prepared only with secondary antibodies (data not shown). Images were captured by using Nikon Eclipse 90i and NIS elements imaging systems, and then processed by Image J (Laboratory for Optical and Computational Instrumentation, Madison, WI, United States).

### Statistics Analysis

All reported data are representative of more than four independent experiments (mean ± S.E.M.). Statistical analysis of gene expression was performed using Graphpad Prism 5.0 (Graphpad Inc., La Jolla, CA, United States) with an independent two-tailed Student’s *t-*test for two-group comparisons, and for differences of more than two groups, analysis of variance (ANOVA) was utilized with *post hoc* test. The Mann–Whitney *U* test was used to analyze the density of immunoblotting bands and the positive cells of immunohistochemistry staining. *p*-values less than or equal to 0.05 were considered as statistically significant.

## Results

### Sonic Hedgehog Was Synthesized by Osteoarthritis Mesenchymal Stromal Cell and Its Receptors Were Highly Expressed by Osteoarthritic Chondrocytes

**FIGURE 1:**
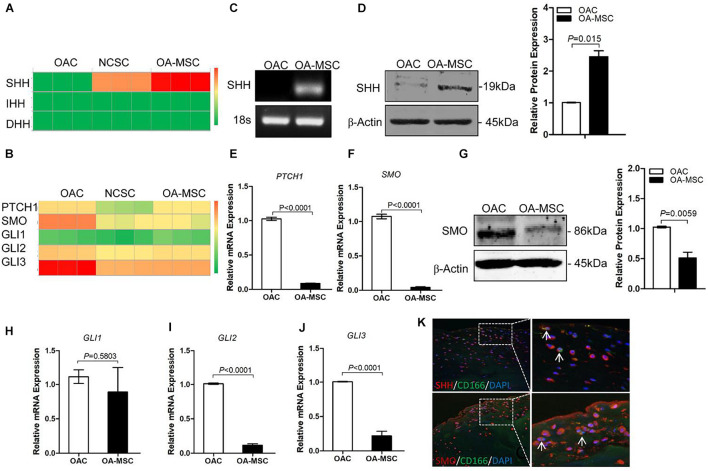
Sonic Hedgehog (*SHH*) was synthesized by human cartilage mesenchymal stromal cell (MSC) while hedgehog (HH) receptors and transcription factors were expressed by human chondrocyte. **(A)** Heat map of copy numbers of transcripts encoding HH ligands (*SHH, IHH, DHH*) in osteoarthritic chondrocytes (OAC), normal cartilage stromal cells (NCSC), and osteoarthritis mesenchymal stromal cell (OA-MSC). **(B)** Heat map of copy numbers of transcripts encoding HH receptors (*PTCH1, SMO*) and transcription factors (*GLI1, GLI2, GLI3*). **(C)** Real-time RT-PCR analysis validated the upregulation of *SHH* transcript in OA-MSC. Total RNA was isolated from primary human OAC and OA-MSC. 18S RNA was used as RT-PCR and loading control. Data are representative of three independent experiments. **(D)** Western blot analysis and quantification indicated SHH protein was abundantly expressed by OA-MSC but not OAC. β-actin was used as Western blot analysis and protein loading control. Molecular weight of proteins was indicated. Relative levels of SHH protein expression in OA-MSC are shown in the bar graph (*n* = 3). Real-time RT-PCR analysis validated the up-regulation of *PTCH1* transcript **(E)** and SMO transcript **(F)** in OAC. Total RNA was isolated from primary human OAC and OA-MSC. 18S RNA was used as a normalizing control (*p* < 0.0001). **(G)** Western blot analysis and quantification indicated SMO protein was down-regulated in OA-MSC. β-actin was used as Western blot analysis and protein loading control. Molecular weight of proteins is indicated. Relative levels of SHH protein expression in OA-MSC are shown in the bar graph (*n* ≥ 4). Real-time RT-PCR analysis validated the expression of GLI1 transcript **(H)**, GLI2 transcript **(I)**, and GLI3 transcript **(J)** in OAC and OA-MSC. Total RNA was isolated from primary human OAC and OA-MSC. 18S RNA was used as a normalizing control (*n* ≥ 4). **(K)** Double immunofluorescence histochemical analysis of human osteoarthritis (OA) articular cartilage with anti-sonic hedgehog antibody (rhodamine red), anti-CD166 (cartilage MSC marker) antibody (fluorescein green), and Hoechst nuclei dye (DAPI blue). SHH protein was distributed in CD166-positive OA-MSC cells which synthesized SHH (arrows). SHH protein was also distributed in CD166-negative OACs which SHH bound. SMO protein was distributed in CD166-negative chondrocytes (arrows). The images shown are representative of multiple tissue samples (*n* = 3). Scale bar = 125 μm.

To delineate HH signaling pathways in human cartilage, we performed RNAseq to quantify the gene expression of HH family members and their receptors in human OA chondrocytes (OAC), normal cartilage stromal cells (NCSC), and OA cartilage mesenchymal stromal cells (OA-MSC). RNA-seq analysis indicated that neither of the three types of cells in human adult articular cartilage expressed Indian Hedgehog (*IHH*) or Desert Hedgehog (*DHH*). Sonic Hedgehog (*SHH*) was expressed by cartilage MSCs but not by OAC. NCSC initiated the expression of *SHH* and its expression was further increased in OA-MSC during aging (Figure 1A). Real-time RT-PCR analysis confirmed that *SHH* was expressed by OA-MSC but not OAC (Figure 1C). Western blot analysis indicated that OA-MSC expressed SHH protein, in contrast to the diminished amount of SHH protein levels in OAC (Figure 1D). Double immuno-fluorescence histochemistry analysis of human OA cartilage indicated SHH protein was co-localized with CD166-positive MSC, where it was synthesized (Figure 1K, upper panels). SHH was also localized in CD166-negative chondrocytes indicating its binding to the OAC (Figure 1K). SHH was present in OAC after OAC was incubated in the OA-MSC conditioned medium (Supplementary Figure 1). RNA-seq analysis indicated that the HH receptors *PTCH1* and *SMO* and transcription factors *GLI2* and *GLI3* were mainly synthesized by OAC (Figure 1B). Real-time RT-PCR analysis validated the finding (Figures 1E,F,H–J). Western blotting analysis indicated that OAC contained a significantly higher level of SMO protein than OA-MSC (Figure 1G). Double immuno-fluorescence histochemistry analysis of human OA cartilage indicated that SMO protein was localized mainly in CD166-negative OAC (Figure 1K, lower panels). These data suggested that SHH, synthesized by NCSC and elevated in OA-MSC, was the major HH signaling molecule in human OA cartilage. Since the HH receptor SMO was present at higher level in OAC and at lower level in OA-MSC, SHH might affect OAC in a paracrine manner and OA-MSC in an autocrine manner.

### Sonic Hedgehog Induced Proliferation, Chondrogenesis, Hypertrophy, and Senescence-Associated Secretory Phenotype in Osteoarthritis Mesenchymal Stromal Cell

**FIGURE 2:**
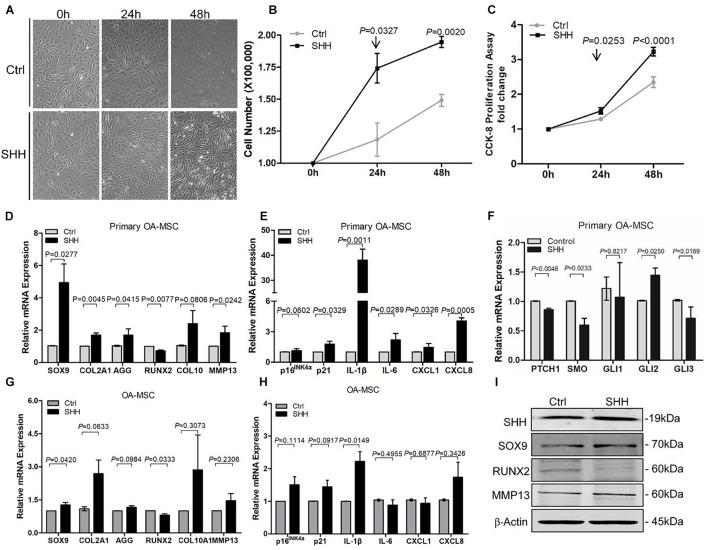
Sonic hedgehog (SHH) promoted osteoarthritis mesenchymal stromal cell (OA-MSC) proliferation and gene expression of the markers for chondrogenesis, hypertrophy, catabolism, senescence, and senescence-associated secretory phenotype (SASP). **(A)** Micrographs of primary OA-MSCs and their treatment with human SHH recombinant protein for 24 and 48 h. Images are representative of cell culture samples in triplicates. Scale bar = 125 μm. **(B)** Cell number change of OA-MSC in response to human recombinant SHH incubation for 24 and 48 h. *n* ≥ 4 individual biological samples. **(C)** Cell proliferation rate of OA-MSC in response to human recombinant SHH incubation for 24 and 48 h. Cell proliferation rate was quantified by the CCK-8 proliferation assay. *n* = 5 biological replicates. Real-time RT-PCR analysis of transcripts encoding markers of chondrogenesis and hypertrophy **(D)**, cell senescence, and SASP **(E)**, hedgehog (HH) signaling **(F)** in primary OA-MSC. 18S RNA was used as a reference gene for RT-PCR amplification. *p*-values were indicated *n* = 3. Real-time RT-PCR analysis of transcripts encoding markers of chondrogenesis and hypertrophy **(G)**, cell senescence, and SASP **(H)**, in OA-MSC cell line. 18S RNA was used as a reference gene for reverse transcriptase-polymerase chain reaction (RT-PCR) amplification. *p*-values were indicated (*n* = 3). **(I)** Western blot analysis of protein levels of SHH, SOX9, RUNX2, and MMP13 in OA-MSC cell line in response to human recombinant SHH treatment. β-actin was used as a protein loading control. Data shown are representative of three biological samples (*n* = 3).

To study the autocrine effects of SHH on OA-MSC, we treated primary MSCs isolated from human OA cartilage as well as OA-MSC cell line with 5 μg/ml human recombinant SHH (N-terminal peptide) (Figure 2A). SHH treatment for 24 h and 48 h significantly increased the cell number (Figure 2B) and the proliferation rate (Figure 2C) of primary MSCs. SHH significantly increased *SOX9* but decreased *RUNX2* transcription factor at both mRNA level (Figures 2D,G) and protein level (Figure 2I). SHH also stimulated chondrogenesis markers *COL2A1* and *ACAN* and hypertrophic markers *COL10A1* and *MMP13* (Figure 2D) in primary OA-MSC. SHH treatment activated *GLI2* expression without inducing the HH receptor *PTCH1* or *SMO* (Figure 2F), suggesting the activation of non-canonical HH signaling independent of HH receptors in OA-MSC. SHH significantly induced *IL-1*β in both primary OA-MSC and OA-MSC cell lines (Figures 2E,H). SHH also significantly induced cell senescence marker p21 and SASPs including *IL6*, *CXCL1*, and *CXCL8* in primary OA-MSC (Figure 2E). Taken together, SHH stimulated cell proliferation, chondrogenesis, hypertrophy, and senescence-associated inflammation in OA-MSCs.

### Sonic Hedgehog Was Required for Osteoarthritis Mesenchymal Stromal Cell Proliferation, Chondrogenesis, Hypertrophy, and Senescence Marker Expression

**FIGURE 3:**
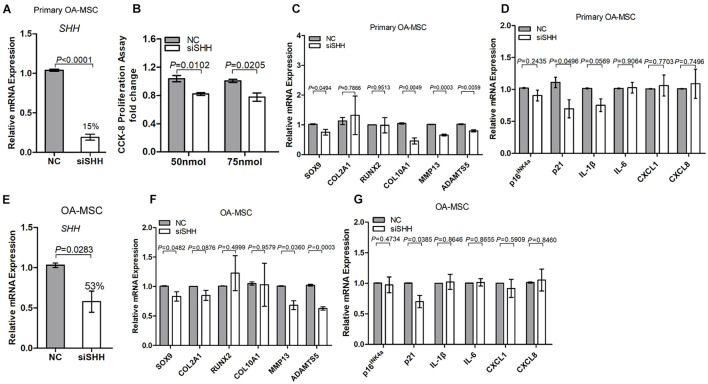
Knocking down sonic Hedgehog (*SHH*) in osteoarthritis mesenchymal stromal cell (OA-MSC) inhibited proliferation and gene expression of markers of chondrogenesis, catabolism, and senescence. **(A)** Significant inhibition of *SHH* mRNA levels by SHH-siRNA (siSHH) transfection in primary OA-MSC. NC: normal control with mock transfection. *p*-value is indicated (*n* = 3). **(B)** Significant inhibition of OA-MSC cell proliferation by siSHH in a dosage-dependent (50 and 75 nmol) manner. After transfection of siSHH for 48 h, the OA-MSC proliferation rate was quantified by CCK-8 proliferation assay. siSHH transfection significantly inhibited *SOX9, MMP13*, and *ADAMTS5* **(C)** and *p21* **(D)** in primary OA-MSC. **(E)** Significant inhibition of *SHH* mRNA levels by siSHH transfection in OA-MSC cell line. NC: normal control with mock transfection. *p*-value is indicated (*n* = 3). siSHH transfection significantly inhibited *SOX9*, *MMP13*, and *ADAMTS5* **(F)** and *p21* **(G)** in OA-MSC cell line. All data are reported as mean ± S.E.M (*n* = 3). *p*-value is indicated.

To determine whether SHH was necessary for OA-MSC proliferation, chondrogenesis, hypertrophy, and senescence, we knocked down *SHH* expression with SHH siRNA (siSHH) (Figures 3A,E). Knocking down *SHH* significantly inhibited the OA-MSC cell proliferation (Figure 3B). It also significantly inhibited the expression of chondrogenesis marker *SOX9*, hypertrophic marker *MMP13*, and senescence marker *p21* in both primary OA-MSC (Figures 3C,D) and OA-MSC cell lines (Figures 3F,G). Therefore, *SHH* was necessary to induce proliferation, chondrogenesis, hypertrophy, and cellular senescence of OA-MSC.

### Sonic Hedgehog Induced Osteoarthritic Chondrocytes Apoptosis, Catabolism, and Senescence-Associated Secretory Phenotype Synthesis

**FIGURE 4:**
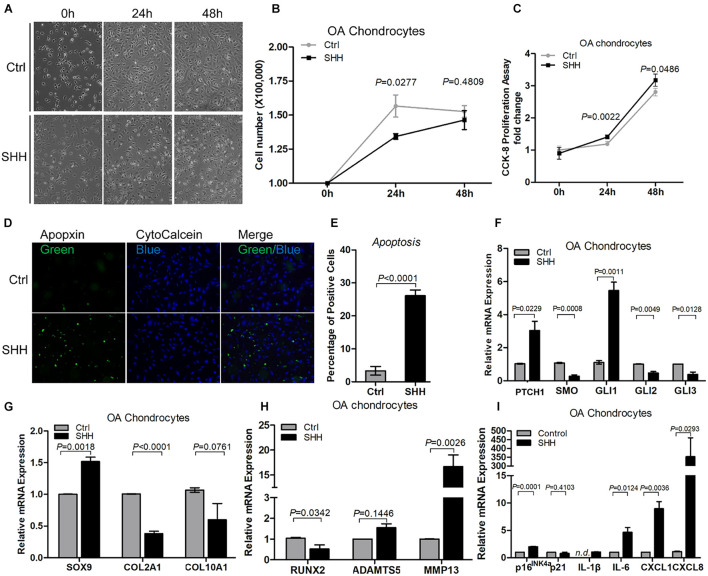
Sonic hedgehog (SHH) induced chondrocyte apoptosis and gene expression of the markers of hedgehog (HH) signaling, catabolism, senescence, and senescence-associated secretory phenotype (SASP) in chondrocytes. **(A)** Micrographs of primary osteoarthritic chondrocytes (OACs) and their treatment with human SHH recombinant protein for 24 and 48 h. SHH treatment induced shrunken cell shape and cell detachment. Images are representative of cell culture samples in triplicates. Scale bar = 125 μm. **(B)** Cell number change of OAC in response to human recombinant SHH incubation for 24 and 48 h. *n* = 4 individual biological samples. **(C)** Cell proliferation rate of OAC in response to human recombinant SHH incubation for 24 and 48 h. Cell proliferation rate was quantified by the CCK-8 proliferation assay. *n* = 5 biological replicates. **(D)** Fluorescence cytochemical analysis of chondrocyte apoptosis induced by human recombinant SHH. Live chondrocytes were stained blue with CytoCalcein Violet 450, while apoptotic cells were stained green with Apopxin green indicator. Scale bars = 125 μm. **(E)** The percentage of OAC apoptosis induced by human recombinant SHH. *n* = 4 individual biological samples. Real-time reverse transcriptase-polymerase chain reaction (RT-PCR) analysis of transcripts encoding markers of HH signaling **(F)** chondrogenesis **(G)**, catabolism **(H)**, and cell senescence and SASP **(I)** in primary OAC. Ctrl, Control (mock treatment).

To determine the effect of SHH on OAC, we treated primary human OA chondrocytes with recombinant SHH (Figure 4A). SHH treatment for 24 h significantly decreased the OAC cell number despite a slight but significant increase of the OAC cell proliferation rate (Figures 4B,C). To determine whether cell death contributed to the decrease of OAC cell number, we quantified OAC cell apoptosis with apopxin labeling. SHH significantly increased OAC cell apoptosis (Figures 4D,E). It also significantly induced *PTCH1* and *GLI1*, suggesting that SHH activated PTCH1 receptor-dependent canonical signaling in OAC (Figure 4F). Similar to OA-MSC, SHH stimulated *SOX9* and inhibited *Runx2* (Figures 4G,H). However, SHH inhibited *COL2A1* and stimulated *MMP13*, suggesting an induction of catabolism in OAC (Figures 4G,H). SHH also induced cellular senescence marker *p16* and SASP genes including *IL-1*β, *IL-6, CXCL1*, and *CXCL8* (Figure 4I).

### Osteoarthritis Mesenchymal Stromal Cell Conditioned Medium Recapitulated the Sonic Hedgehog Effects on Osteoarthritic Chondrocytes Catabolism and Apoptosis

**FIGURE 5:**
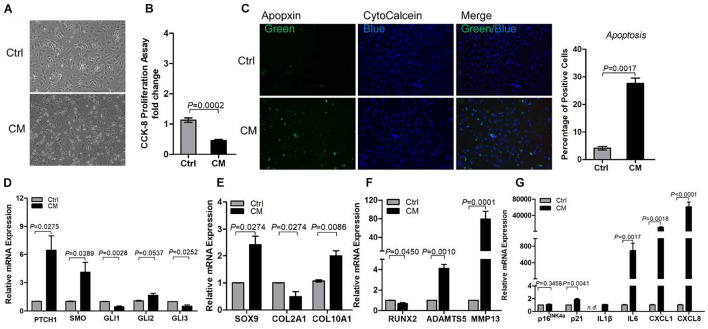
Osteoarthritis mesenchymal stromal cell (OA-MSC)-derived conditioned medium (CM) stimulated chondrocyte apoptosis and expression of catabolic and senescence-associated secretory phenotype (SASP) genes in osteoarthritis (OA) chondrocytes. **(A)** Micrograph of osteoarthritic chondrocytes (OAC) shrunken cell morphology and detachment induced by OA-MSC-derived CM. **(B)** Inhibition of OAC cell proliferation by OA-MSC-derived CM. Cell proliferation rate was quantified by CCK-8 proliferation assay. **(C)** Fluorescence cytochemical analysis of chondrocyte apoptosis induced by OA-MSC-derived CM. OA chondrocytes were treated with OA-MSC CM at 37°C, 5% CO_2_ for 24 h. Live chondrocytes were stained blue with Cytocalcein violet 450, while apoptotic cells were stained green with Apopxin green indicator. Scale bars = 125 μm. Five individual images taken from three different human chondrocyte samples were assessed by Image J software and analyzed by two-tailed non-paired Students’ *t-*test. Real-time RT-PCR analysis of transcripts encoding markers of HH signaling **(D)**, chondrogenesis and hypertrophy **(E)**, catabolism **(F)**, and cell senescence and SASP **(G)**, in primary OAC treated with OA-MSC CM for 24 h. 18S RNA was used as a reference gene for RT-PCR amplification. *p*-values are indicated (*n* = 3). Results are presented as mean ± S.E.M. from at least four independent experiments.

To understand the paracrine regulation of OAC by OA-MSC, we incubated OAC with the OA-MSC conditioned medium (Figure 5A). The OA-MSC conditioned medium significantly inhibited OAC cell proliferation (Figure 5B) and induced OAC cell death *via* apoptosis (Figures 5C,D). The OA-MSC conditioned medium induced HH receptor *PTCH1* expression in OAC (Figure 5D), indicating that the OA-MSC conditioned medium activated HH signaling in OAC. Similar to the effects of SHH, the OA-MSC conditioned medium increased *SOX9* and inhibited *Runx2* in OAC (Figures 5E,F). The OA-MSC conditioned medium treatment also decreased *COL2A1* but increased *MMP13* (Figures 5E,F). It also induced senescence marker *p21* and SASP genes including *IL1B, IL6, CXCL1*, and *CXCL8* in OAC (Figure 5G).

### The Paracrine Effects of Osteoarthritis Mesenchymal Stromal Cell on Osteoarthritic Chondrocytes Apoptosis and Catabolism Depended on Sonic Hedgehog Activity

**FIGURE 6:**
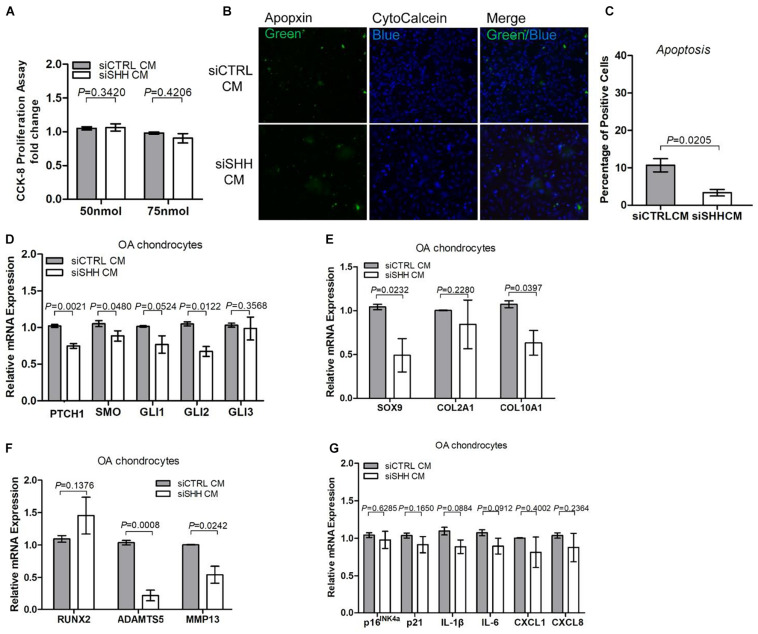
Knocking down sonic hedgehog (SHH) in osteoarthritis mesenchymal stromal cell (OA-MSC)-derived conditioned medium inhibited chondrocyte apoptosis and expression of hedgehog (HH) signaling and catabolic genes in osteoarthritic chondrocytes (OAC). **(A)** Knocking down SHH in OA-MSC-derived conditioned medium did not affect OAC proliferation. OA-MSC-derived conditioned medium was collected from OA-MSC transfected with SHH-siRNA (siSHH CM) or scrambled control siRNA (siCTRL CM). Two dosages of siRNA (50 and 75 nmol) were tested. OACs were incubated in the conditioned medium for 24 h. **(B)** Knocking down SHH in OA-MSC inhibited OAC apoptosis induced by OA-MSC CM. Fluorescence cytochemical analysis of chondrocyte apoptosis induced by OA-MSC-derived CM (siCTRL CM and siSHH CM). Alive chondrocytes were stained blue with Cytocalcein violet 450, while apoptotic cells were stained green with Apopxin green indicator. Scale bars = 125 μm. **(C)** Quantification of the apoptosis rate of OAC incubated in siCTRL CM and siSHH CM derived from OA-MSC. *p*-value is indicated (*n* = 3). Real-time reverse transcriptase-polymerase chain reaction (RT-PCR) analysis of transcripts encoding markers of HH signaling **(D)**, chondrogenesis and hypertrophy **(E)**, catabolism **(F)**, and cell senescence and senescence-associated secretory phenotype (SASP) **(G)**, in primary OAC treated with siCTRL Cm and siSHH CM for 24 h. 18S RNA was used as a reference gene for RT-PCR amplification. *p*-values are indicated (*n* = 3).

To determine whether the effects of the OA-MSC conditioned medium were due to SHH, we knocked down *SHH* by transfecting OA-MSC with SHH siRNA (siSHH). The conditioned medium of *SHH*-knocked down (siSHH-CM) OA-MSC cells was collected for treatment of OAC. Knocking down *SHH* in OA-MSC did not have any effect on OAC cellular proliferation (Figure 6A). This suggested that the inhibitory effect of the OA-MSC conditioned medium on OAC cellular proliferation observed previously (Figure 5B) was not due to the SHH in the OA-MSC conditioned medium. On the other hand, the conditioned medium of *SHH* knocked-down OA-MSC significantly inhibited OAC apoptosis (Figures 6B,C), suggesting that SHH was critical for the paracrine effect of OA-MSC to induce OAC apoptosis. The expression of HH receptors *PTCH1* and *SMO* and transcription factor *GLI2* was significantly inhibited by siSHH-CM (Figure 6D), confirming that the HH signaling in OAC was inhibited by the *SHH* knock-down conditioned medium. The conditioned medium of *SHH* knocked-down OA-MSC significantly inhibited expression of *SOX9*, *ADAMTS5*, and *MMP13* (Figures 6E,F), indicating that *SHH* was necessary for stimulation of expression of these genes by the OA-MSC conditioned medium, as observed previously (Figures 5E,F). On the other hand, the *SHH* knock-down OA-MSC conditioned medium did not affect the expression of cellular senescence markers or SASP gene expression in OAC (Figure 6G), suggesting that other factors in the OA-MSC conditioned medium were sufficient to induce cellular senescence and SASP in OAC.

## Discussion

In this study, we show that SHH is synthesized by OA cartilage mesenchymal stromal cells and plays an important role in regulating MSC growth, replicative senescence, and chondrocyte catabolism as well as apoptosis during OA pathogenesis. Previously, SHH has been shown to be a key molecule to regulate embryonic morphogenesis of skeletal tissues including meniscus (Wei et al., 2021), tendon enthesis (Fang et al., 2020), and limb bud (Alman, 2015). Synthesized by mesenchymal progenitor cells, SHH affects different types of cells located nearby to regulate tissue morphogenesis, maintain organ homeostasis, and drive tissue aging (Ho et al., 2009; Lin et al., 2014; Ali et al., 2019). SHH regulates progenitor cell proliferation and differentiation in a concentration-dependent manner (Lin et al., 2014). In adult, SHH acts as an oncogene to transform adult stem cells to cancer stem cells and is involved in tumorigenesis of many types of cancer (Ali et al., 2019; Zhu et al., 2020). However, aside from cancer, the role of SHH in adult human tissue degeneration and aging-associated degenerative diseases was not well documented. SMO, a hallmark of HH signal transduction, has been shown to be within the cell senescence signature transcriptome core (Hernandez-Segura et al., 2017; Grandi et al., 2020; Yousefzadeh et al., 2021). However, the role of the HH pathway in cell senescence was not understood.

We demonstrate here that adult human OA articular chondrocytes (OAC) express the HH receptors *PTCH1* and *SMO* but do not express any of the HH ligands including *SHH*, *IHH*, and *DHH*. It is known that, in the absence of HH ligand, PTCH1 suppresses HH signaling by binding to SMO (Alman, 2015). In the presence of HH ligand, HH binds to PTCH1 and activates HH signaling by releasing PTCH1 binding to SMO (Lin et al., 2009). Our observation suggests that, in the absence of any HH ligand, PTCH1 suppressed HH signaling and kept OAC in a quiescent state. We found that expression of SHH, but not IHH or DHH, was initiated in normal cartilage stromal cells (NCSC). This suggests that NCSC may initiate cellular proliferation and chondrogenesis by providing SHH ligand to the neighboring chondrocytes that express HH receptors. Intriguingly, we found that the expression level of SHH was further increased in the cartilage MSC of aged OA patients. This suggests that SHH might play a role in aging-associated OA.

To define the mechanistic basis of HH signaling in OA cartilage degeneration, we treated OA-MSC with SHH in an autocrine manner and OAC in a paracrine manner. For OA-MSC treatment, we used both primary OA-MSC and OA-MSC cell lines. Primary MSCs were isolated from human OA cartilage with fibronectin adhesion assay, since MSCs expressed high levels of fibronectin receptor CD49e (Jayasuriya et al., 2018). The primary MSCs in OA cartilage (primary OA-MSC) comprised heterogeneous MSC populations containing the proliferative and chondrogenic NCSC-like MSC and the senescent and osteogenic MSCs (Ji et al., 2019; Grandi et al., 2020). We also tested an OA-MSC cell line, which was derived from a single colony of primary human OA-MSCs (Liu et al., 2020). It showed that OA-MSC expressed very low levels of HH receptors and transcription factors in comparison to OAC. Furthermore, SHH-induced HH signaling activated in the OA-MSCs without inducing the *PTCH1* expression. These data suggest that SHH activated non-canonical signaling in OA-MSC.

**FIGURE 7:**
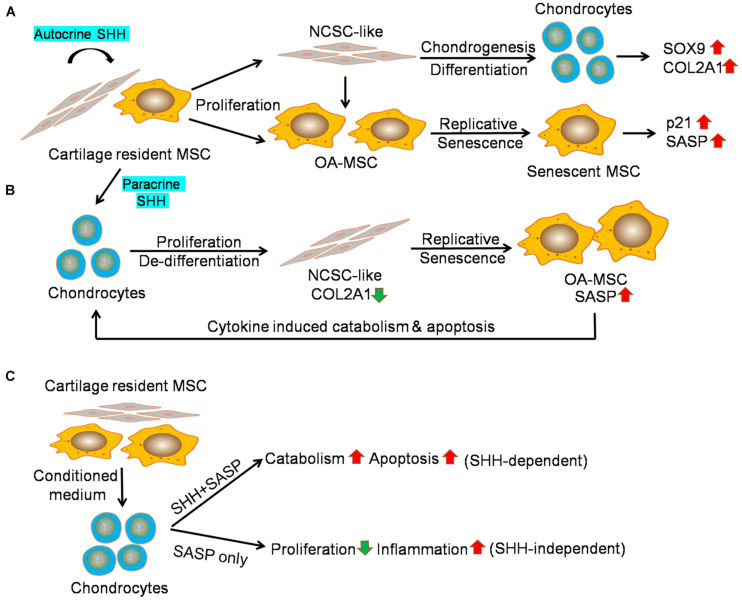
Sonic hedgehog (SHH) mediates interaction of mesenchymal stromal cell (MSC) and chondrocytes in human osteoarthritis (OA) articular cartilage. **(A)** Working hypothesis of the effect of autocrine SHH on cartilage MSC. Cartilage MSC contains heterogeneous populations of NCSC-like MSC and senescent OA-MSC. SHH stimulates proliferation of NCSC-like MSC and induces chondrogenic differentiation. It results in the elevation of chondrogenesis markers including *SOX9* and *COL2A1*. SHH also stimulates proliferation of OA-MSC and induces replicative senescence. It results in the elevation of cell senescence markers including *p21* and SASP *IL1B*. **(B)** Working hypothesis of the effect of paracrine SHH on chondrocytes. SHH stimulates proliferation and de-differentiation of OA chondrocytes. It results in a decrease of expression of chondrocyte marker *COL2A1*. SHH also induces replicative senescence of cartilage MSC. It results in the elevation of cell senescence markers including pro-inflammatory cytokines. Upon binding to the receptors, cytokines induced catabolism and apoptosis in OA chondrocytes. **(C)** Working hypothesis of the effect of OA-MSC on OAC revealed by the conditioned medium experiment data. The OA-MSC conditioned medium inhibited chondrocyte proliferation and induced catabolism, apoptosis, and inflammation in chondrocytes. While chondrocyte catabolism and apoptosis depend on OA-MSC-derived SHH, inhibition of chondrocyte proliferation and stimulation of chondrocyte inflammation are SHH independent. SHH may regulate these events indirectly through inducing OA-MSC cell senescence and senescence-associated secretory phenotype (SASP) production.

SHH treatment of OA-MSC induced not only proliferation and chondrogenesis but also markers of cellular senescence and SASP genes including *p21, IL1B, IL6, CXCL1*, and *CXCL8*. It is striking that SHH stimulated *IL1B* for more than 35-fold in primary OA-MSC. This SHH regulatory pattern was mostly recapitulated in the OA-MSC cell line, although the extent of induction with the same concentration of SHH was not as high as in the primary OA-MSC. One possible reason could be due to the fact that primary OA-MSC contained NCSC-like MSCs in OA cartilage, which were co-purified by the fibronectin adhesion assay (Fellows et al., 2017; Liu et al., 2020). The OA-MSC cell line, on the other hand, was derived from a single colony containing more uniformed cells. The NCSC-like MSCs could exhibit a more dynamic reaction in response to SHH treatment since they expressed lower levels of *SHH*, cellular senescence markers, and SASP genes to begin with. SHH treatment could also exert different effects on the NCSC-like MSC and the more senescent OA-MSC. While SHH stimulated proliferation and chondrogenesis in the NCSC-like MSC, it might drive replicative senescence and SASP expression in the more senescent OA-MSC (Figure 7A). SHH ligand was essential to the activities of both NCSC-like MSC and senescent OA-MSC, since *SHH* knockdown suppressed not only proliferation and chondrogenesis but also the expression of senescence markers and SASP genes in primary OA-MSC. SHH knockdown also suppressed gene expression of the markers of chondrogenesis, senescence, and catabolism in the OA-MSC cell line, although to a lesser extent than the suppression in the primary OA-MSC.

We demonstrated that SHH, synthesized by OA-MSC, regulated OAC in a paracrine manner. OAC expressed HH receptors and transcription factors. SHH treatment further induced *PTCH1* levels in OAC. This suggests that SHH activated HH canonical signaling in OAC. To our surprise, SHH induced chondrocyte apoptosis despite its stimulating effects on proliferation. This resulted in a net decrease of the chondrocyte number due to cell death. The reduction of chondrocyte number might explain the decrease of *COL2A1*, which was synthesized by chondrocytes. However, SHH treatment of OAC increased *SOX9*, which was also synthesized by chondroprogenitor/NCSCs (Jayasuriya et al., 2019). This suggests that SHH stimulated dedifferentiation of chondrocytes into NCSC-like cells, which expressed *SOX9*. The reduction of the chondrocyte marker *COL2A1* was also consistent with chondrocyte dedifferentiation induced by SHH (Lin et al., 2014). In addition to the MSC that resulted from SHH-induced OAC dedifferentiation, primary OAC preparation from human OA cartilage also contained a very small percentage of OA-MSC (Jayasuriya et al., 2019; Liu et al., 2020). Since SHH treatment of OA-MSC induced SASP expression, it might contribute to the increase of SASP gene expression in the primary OAC culture. Because the receptors of SASP/pro-inflammatory cytokines such as IL-1R were mainly expressed in OAC but not in OA-MSC (Liu et al., 2021), SHH induction of inflammatory cytokines would induce apoptosis of OAC but not OA-MSC (Figure 7B).

We also demonstrated that SHH played an important role in the OA-MSC/OAC interaction. When we incubated OAC with OA-MSC conditioned medium (CM), OA-MSC CM induced *PTCH1*. This indicated that OA-MSC CM contained functional SHH ligand. OA-MSC CM recapitulated most of the effects of SHH when incubated with OAC including the increase of chondrocyte apoptosis, stimulation of *SOX9*, and induction of catabolic markers including *ADAMTS5* and *MMP13*. Conversely, incubation of OAC with OA-MSC CM in which SHH had been knocked-down reversed these effects. Thus, SHH was both necessary and sufficient for OA-MSC induction of OAC catabolism and apoptosis (Figure 7C).

Our data also indicated that some of the effects of OA-MSC CM on OAC were not mediated by SHH directly. While SHH stimulated OAC proliferation, OA-MSC CM inhibited OAC proliferation. The OA-MSC CM in which SHH had been knocked down did not change the OAC proliferation rate. This suggests that the OA-MSC inhibition of OAC proliferation was not mediated by SHH. Since the OA-MSC CM contained high concentrations of SASPs (Liu et al., 2021), the inhibition of chondrocyte proliferation could be achieved by pro-inflammatory cytokines such as IL-1β as demonstrated previously (Fellows et al., 2017; Grandi et al., 2020). Knocking down SHH in OA-MSC CM did not inhibit the SASP gene expression in OAC. This was consistent with the observation that SASPs were mainly synthesized by OA-MSC but not by OAC (Liu et al., 2021).

In summary, we show that SHH plays an important role in mediating the interaction between MSC and chondrocytes in human cartilage. SHH is made by MSC and increases in senescent OA-MSC during aging. SHH stimulates MSC growth, senescence, and SASP production, and accounts for the induction of chondrocyte catabolism and apoptosis by OA-MSC during OA pathogenesis. The findings suggest that SHH is a key signaling molecule to mediate cell interaction in aged tissues that results in aging-associated tissue degeneration, and that SHH should be considered as a molecular target for OA therapy. SHH-targeted therapy for OA treatment is consistent with the therapeutic strategy of treating cancers caused by SHH over-activation. One potential shortcoming of this study is that the experiments were performed in 2D culture *in vitro* since we utilized human tissues and cells. However, it may pave the way for testing the role of SHH mediating the OA-MSC/OAC interaction during OA pathogenesis in animal models *in vivo*.

## Data Availability Statement

The data presented in the study are deposited in the NCBI repository, accession number GSE176199 .

## Ethics Statement

The studies involving human participants were reviewed and approved by the IRB Committee of Rhode Island Hospital. The patients/participants provided their written informed consent to participate in this study.

## Author Contributions

MF: conception and design of this study, acquisition and interpretation of the data, curation of the data, drafting the manuscript or critically revising the manuscript, and approved the final version of the manuscript. WL and JD: acquisition and interpretation of the data and approved the final version of the manuscript. YQ: drafting the manuscript or critically revising the manuscript and approved the final version of the manuscript. QC: conception and design of this study, acquisition and interpretation of the data, drafting the manuscript or critically revising the manuscript, and approved the final version of the manuscript. All authors contributed to the conception and design of this study, acquisition and interpretation of data, and drafting the article or critically revising the manuscript, and approved the final version of the manuscript.

## Conflict of Interest

QC is a co-inventor of a pending patent application of generating OA-MSC cell lines. OA-MSC cell lines have been licensed to and available at Applied Biological Materials Inc. (ABM). The remaining authors declare that the research was conducted in the absence of any commercial or financial relationships that could be construed as a potential conflict of interest.

## Publisher’s Note

All claims expressed in this article are solely those of the authors and do not necessarily represent those of their affiliated organizations, or those of the publisher, the editors and the reviewers. Any product that may be evaluated in this article, or claim that may be made by its manufacturer, is not guaranteed or endorsed by the publisher.

## Abbreviations

OAC: Osteoarthritic chondrocytes
NCSC: Normal cartilage stromal cells
OA-MSC: Osteoarthritis mesenchymal stromal cell
HH: Hedgehog
IHH: Indian Hedgehog
SHH: Sonic Hedgehog
DHH: Desert Hedgehog
PTCH1: Patched1
SMO: Smoothened
GLI1, GLI2, GLI3: Glioma-associated oncogene 1, 2, 3
RT-PCR: Reverse transcriptase-polymerase chain reaction
SASP: Senescence-associated secretory phenotype
SOX9: Sex-determining region Y-type high mobility group box9.
