# Supplementary material for: Sonic Hedgehog Induces Mesenchymal Stromal Cell Senescence-Associated Secretory Phenotype and Chondrocyte Apoptosis in Human Osteoarthritic Cartilage
Source: Front Cell Dev Biol. 2021 Sep 27;9:716610. doi: 10.3389/fcell.2021.716610 (PMC8502980; doi:10.3389/fcell.2021.716610)
Supplement: Supplementary file 1 [file Image_1.pdf]

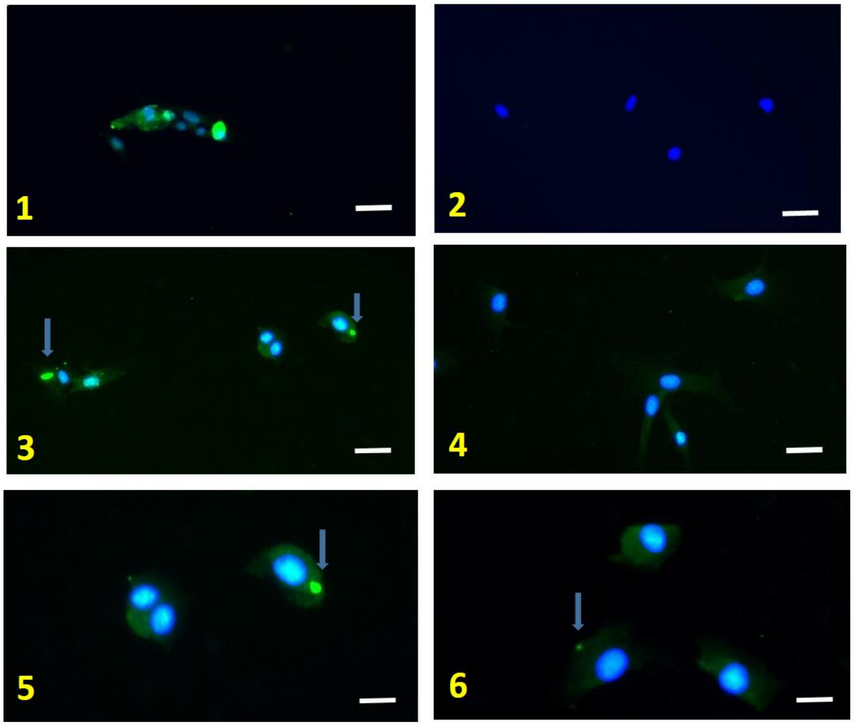

Supplementary Figure 1

Immunofluorescence staining of SHH in human OAC incubated with or without OA-MSC conditioned medium for 24 hrs. **Panel 1** shows the positive green fluorescence staining of SHH in OA-MSC with a primary antibody against SHH. It serves as a positive control for SHH staining. The nucleus was stained blue with the DAPI dye. **Panel 2** shows the negative staining of SHH in OA-MSC without the SHH primary antibody. **Panel 3** shows SHH in the cytoplasm of OAC after incubation in the OA-MSC conditioned medium for 24 hrs. **Panel 4** shows the negative intracellular staining of SHH in the absence of OA-MSC medium. **Panel 5 and 6** provide a close-up image (20X) of the intracellular staining of SHH in OAC incubated in the OA-MSC conditioned medium. Arrows point to the cytoplasmic punctate staining of endocytosed SHH in OAC incubated with OA-MSC conditioned medium. The images are representative of three triplicate of OAC cell culture Immunofluorescence staining. Scale Bar in Panel 1-4 (10X) = 40µm; Scale Bar in Panel 5-6 (20X) = 20µm.
